# Editorial: Cuproptosis and tumors, volume II: from basic research to clinical translation

**DOI:** 10.3389/fcell.2025.1773248

**Published:** 2026-01-13

**Authors:** Bing Liu, Cheng Wang, Jiannan Liu, Chun Xu, Lin-Lin Bu

**Affiliations:** 1 State Key Laboratory of Oral and Maxillofacial Reconstruction and Regeneration, Key Laboratory of Oral Biomedicine Ministry of Education, Hubei Key Laboratory of Stomatology, School and Hospital of Stomatology, Wuhan University, Wuhan, Hubei, China; 2 Department of Oral and Maxillofacial - Head Neck Oncology, School and Hospital of Stomatology, Wuhan University, Wuhan, Hubei, China; 3 Department of Oral and Maxillofacial Surgery, Hospital of Stomatology, Guanghua School of Stomatology, Sun Yat-sen University, Guangzhou, China; 4 Department of Oral Maxillofacial-Head and Neck Oncology, Shanghai Ninth People’s Hospital Affiliated to Shanghai Jiao Tong University School of Medicine, Shanghai, China; 5 Sydney Dental School, Faculty of Medicine and Health, The University of Sydney, Camperdown, NSW, Australia

**Keywords:** cancer metastasis, cuproptosis, diagnosis, nanomedicine, regulated cell death

Copper is an essential trace element involved in mitochondrial metabolism, redox regulation, and enzymatic activity, and its homeostasis is critical for cell survival (Li et al., 2022). Cuproptosis is a newly defined form of regulated cell death (RCD) based on copper ion overload, whose mechanism differs from other RCDs such as apoptosis, necroptosis, pyroptosis, and ferroptosis (Tsvetkov et al., 2022). Copper mediates cuproptosis by directly binding to acyl-transferase enzymes in the tricarboxylic acid cycle, triggering protein aggregation and destabilizing iron-sulfur clusters. Extensive research has demonstrated that cuproptosis is closely linked to tumor metabolism and progression, positioning this pathway as a potential target for cancer mechanism studies and therapeutic interventions (Zhou et al., 2025).

Against this backdrop, the Research Topic was conceived to delve into the emerging biological mechanisms of cuproptosis in cancer and advance its clinical applications. Building upon the findings of Volume I, this volume compiles multiple original research papers that delve into the expression patterns of cuproptosis-related genes (CRGs) and molecules across various cancer types, including oral squamous cell carcinoma (OSCC), colorectal cancer (CRC), colorectal adenocarcinoma (COAD), cervical cancer, and rectal adenocarcinoma. Particular emphasis is placed on analyzing their critical roles in early diagnosis, prognostic evaluation, and therapeutic strategies.

Cuproptosis, as a newly discovered mechanism of RCD, has drawn widespread attention to the molecular mechanisms of its associated genes in various cancers. Liu et al. integrated multiple techniques including single-cell transcriptomics, spatial transcriptomics, and immunohistochemistry, to identify a malignant cell subtype in OSCC exhibiting metal-dependent cell death resistance (MCDR) (Liu et al.). This subtype is strongly associated with lymph node metastasis and treatment resistance, providing a potential biological biomarker for early prediction of lymph node metastasis and precision therapy. Zhuang et al. integrated multi-center clinical data, bioinformatics analysis, and *in vitro/in vivo* functional experiments to reveal that the CRG-ACAD8 is significantly downregulated in metastatic CRC and suppresses tumor metastasis. It exerts its anticancer effects by regulating copper ion-related pathways, immune infiltration, and chemotherapy sensitivity, providing a potential molecular target for the diagnosis, prognostic assessment, and precision treatment of colorectal cancer metastasis (Zhuang et al.). Ye et al. constructed a prognostic risk model based on multiple CRGs (*ORC1*, *PTTG1*, *DLAT*, *PDHB*), revealing the central role of cuproptosis in metabolic reprogramming, immune microenvironment modulation, and immunotherapy response, and further confirmed the therapeutic potential of inducing cuproptosis through the FDX1-elesclomol axis in COAD (Ye et al.). In cervical cancer, Cui et al. demonstrated that cuproptosis also plays an important biological role. By establishing a cuproptosis-related molecular classification and functionally validating FOXJ1, they confirmed that FOXJ1 could promote cuproptosis while suppressing tumor cell invasion, migration, and epithelial-mesenchymal transition (Cui et al.). Addressing the long-standing lack of systematic cuproptosis research in rectal adenocarcinoma, Li et al. integrated multi-omics data to establish a cuproptosis-related prognostic model, revealing the involvement of cuproptosis in tumor immune regulation and drug sensitivity (Li et al.). Across different tumor entities, researchers have explored how CRGs correlate with patient survival, tumor microenvironment composition, and immune cell infiltration, offering new insights into the interplay between metabolic cell death pathways and antitumor immunity. Some studies have further developed predictive models and risk scoring systems based on cuproptosis-related signatures, offering innovative tools for patient stratification and treatment decision-making.

Despite this, the role and mechanisms of cuproptosis in various tumor types remain to be further explored. By synthesizing these studies, this Research Topic aims to advance the in-depth investigation of cuproptosis’s role in tumor biological mechanisms and to facilitate the translation of its mechanisms into tumor precise therapeutic strategies. This Research Topic and existing evidence have indicated that cuproptosis holds significant potential for cancer metastasis diagnosis and treatment. CRGs not only serve as a potential biomarker for early diagnosis and prognostic assessment of cancer metastasis, but cuproptosis-based nanomedicines have also been developed for precision synergistic anti-metastatic therapy (Li et al., 2025). The core objective of this Research Topic is to deepen our understanding of the mechanisms linking cuproptosis and tumor, thereby paving the way for novel approaches to tumor diagnosis, prognosis assessment, and treatment through the exploitation of cuproptosis-related mechanisms.
